# Impacts of chemical fertilizer reduction on grain yield: A case study of China

**DOI:** 10.1371/journal.pone.0298600

**Published:** 2024-03-07

**Authors:** Changjiang Xiong, Xianghao Zhao

**Affiliations:** 1 Institute of Finance and Economics, Shanghai University of Finance and Economics, Yangpu District, Shanghai, P. R. China; 2 College of Economics, Xinjiang University of Finance and Economics, Urumqi, P. R. China; The Ohio State University, UNITED STATES

## Abstract

Reducing fertilizer usage is a crucial measure for achieving high-quality development in Chinese agriculture. Utilizing panel data from 31 Chinese provinces spanning from 2004 to 2019, this study empirically analyzes the dynamic relationship between fertilizer application and grain production, exploring the underlying mechanisms. The study findings reveal that the application of fertilizers maintains a positive impact on grain production. The two variables will demonstrate a dynamic alternation between "strong decoupling" and "retreat decoupling," suggesting that grain production may either increase or gradually decline, while fertilizer application exhibits a decreasing trend. Mechanism analysis reveals a distinct substitution relationship between fertilizer use efficiency and application quantity. Increasing fertilizer use efficiency while reducing application quantity still facilitates the stable and increased production of grains. Heterogeneity analysis indicates that the efficiency of fertilizer use has a more pronounced impact on grain yield in the eastern and western regions. Increasing fertilizer quantity is detrimental to wheat yield but has a promoting effect on corn yield. However, in the main grain-producing areas, increasing fertilizer quantity can enhance wheat yield but is unfavorable for the overall grain yield. Additionally, nitrogen fertilizer input has exceeded the optimal level compared to potassium fertilizer. Continuously increasing nitrogen fertilizer input will hinder the increase in grain yield. Therefore, there is a need to shift from the notion of "more fertilizer is better" and focus on improving fertilizer use efficiency to transition from the emphasis on "quantity" to "quality" of fertilizer application.

## 1. Introduction and literature review

The contribution rate of fertilizer to grain production increasement in China was once close to 50% [1]. On average, every kilo of fertilizer (pure nutrient) input can increase grain production by 7.5kg [2], which is an important guarantee for food security. However, the excessive use of fertilizer has seriously restricted China’s economic growth and ecological environment protection [3], becoming the “Sword of Damocles” of agricultural production. The research shows that agricultural non-point source pollution caused by fertilizer and other chemicals directly leads to the loss of 0.5%~1% of China’s GDP [4], and the cultivated land fertility decreases by 0.25 percentage points annually on average, which is 10–20 percentage points lower than that of western developed countries [5]. Moreover, the excessive use of fertilizer has caused the overproof of the content of nitrate, heavy metals and other harmful elements in groundwater as well as crops leading to food borne diseases that are increasingly endangering people’s health and life [6, 7]. Therefore, the No. 1 Central Document in 2015 clearly pointed out that efforts should be intensified to control pollution from non-point agricultural sources, and issued a series of documents called “Action Plan for Zero Growth of Fertilizer Use by 2020” (hereinafter referred to as “Action Plan”) attaching great importance to fertilizer reduction production and achieving phased results: China achieved the target of zero growth in the use of chemical fertilizer within two years, and after five years of implementation, the utilization rate of chemical fertilizer for rice, corn and wheat increased from about 35.2% in 2015 to 40.2% in 2020. The discount stock of agricultural fertilizer application decreased from more than 60.22 million tons in 2015 to 54.036 million tons in 2020, recording negative growth for five consecutive years.

However, it is worth noting that with the introduction of the *Action Plan*, on the one hand, the fertilizer input declined in 2016 for the first time since 2004. Was the trend of grain production growth stopped due to fertilizer reduction? On the other hand, in the case of continued negative growth in fertilizer use, although China’s grain production failed to achieve the thirteenth consecutive growth, but the total grain production continued to stabilize at about 650 million tons, achieving the goal of continuous good harvest and effectively guaranteeing the supply of grain. Does this in turn mean that fertilizer reduction does not have a negative impact on food production? In other words, the following questions need to be answered: What are the driving factors of our continuous grain harvest? And how to understand and clarify the logical relationship between fertilizer reduction and grain increase, especially how to clarify the dynamic role of fertilizer in food production? Obviously, the answers to the above questions are not only related to the stability and increase of grain production, but also to the management of surface pollution, which is of great significance to how to promote the reduction of chemical fertilizers in China. Research has shown that although China’s fertilizer input has achieved a statistical reduction and its growth rate has been effectively controlled, it is still necessary to be aware of the rebound in its use [8]. Exploring innovative ways and means to reduce fertilizer is still the key direction that requires for attention. In view of this, the academia has conducted research on how to achieve fertilizer reduction from different perspectives.

At the micro level, scholars mainly analyzed from the perspectives of risk aversion, organic fertilizer substitution and farmland scale. Li and Shang (2021) showed that farmers with different risk aversion awareness had different fertilization behaviors, while farmers with high risk aversion awareness were more inclined to increase fertilizer input to reduce the risk of production reduction [9]. And social norms could directly or indirectly promote farmers to take environmental-friendly measures such as reducing fertilizer, so as to curb the overuse of fertilizer due to risk aversion [10]. Substituting inorganic fertilizers with organic fertilizers is one of the main measures to reduce chemical fertilizer input. The application of organic fertilizer can significantly enhance the absorption of nitrogen, phosphorus, potassium and other nutrients by crops [11, 12]. However, in actual agricultural production, the number of farmers adopting organic fertilizers to replace chemical fertilizers is significantly low [13]. The challenges of applying organic fertilizers are mainly the following: First, the nutrient content and effectiveness of organic fertilizers are not stable [14]. Livestock and poultry manure are crucial sources for producing organic fertilizer, with the global nitrogen content in livestock and poultry manure being equivalent to the total nitrogen use in chemical fertilizers [15]. However, due to nutrient losses and low rates of incorporation into cultivated fields, the actual amount applied to farmland is only 30% of the nitrogen found in chemical fertilizers [16]. Second, the promotion cost of organic fertilizer is higher. In comparison to the simplicity and efficiency of chemical fertilizers, the application of organic fertilizer is notably more labor-intensive and time-consuming. The quantity of organic fertilizer applied per unit area is much higher than that of chemical fertilizer [17]. Third, the long-term application of organic fertilizer may lead to potential pollution issues. Livestock and poultry manure are essential raw materials for producing organic fertilizers. However, in some large and medium-sized poultry and livestock farms, the feed often contains elevated levels of heavy metals beyond the relevant standards [18]. To optimize the use of organic fertilizers, scholars suggest integrating organic agriculture with conventional agriculture, harmonizing the balance between soil organic nutrients and inorganic nutrients. This approach aims to produce more agricultural products with minimal environmental cost [19]. On the other hand, expanding the scale of agricultural land can also increase the proportion of organic fertilizer used by farmers [20]. Further analysis by Zhang and Luo (2020) indicated that larger-scale farmers are more advantageous for the utilization of organic fertilizer [21].

At the macro level, scholars have analyzed the relationship between fertilizer application and grain production from the perspectives of subsidy policy, planting structure and agricultural land property rights. While long-term financial support from the national treasury for agriculture is beneficial for increasing grain production, it can also influence farmers’ fertilization behavior. Providing subsidies to farmers through price support may inadvertently stimulate increased chemical fertilizer usage, leading to environmental issues [22]. However, subsidizing organic fertilizers could encourage farmers to choose organic alternatives, thereby achieving a reduction in chemical fertilizer usage [23]. Wu et al. [24] pointed out that one of the main reasons for the significant reduction on fertilizer is the change of planting structure. The reduction of corn proportion in grain crops has reduced the amount of fertilizer. Yao [25] argued that unstable land tenure exacerbates the application of chemical fertilizers by farmers. The reason is that unstable land tenure fails to guarantee stable land returns for farmers. In order to gain more profits, farmers may intensify the "exploitation" of land without considering the future changes in land quality. Zhou and Wang [26] confirmed this view from the perspective of agricultural land ownership confirmation. Agricultural land ownership confirmation will promote farmers to reduce fertilizer intensity, and promote the use of organic fertilizers and other behaviors conducive to the protection of cultivated land quality.

Furthermore, it is widely acknowledged in the academic community that the strategy of increasing grain production through elevated chemical fertilizer inputs is no longer sustainable. Instead, enhancing the efficiency of chemical fertilizer application is considered a potential driving force for promoting grain production in China. Cai and Tao [27] used the stochastic frontier model to measure the fertilizer efficiency in China over the past 20 years. They found that the overall level of fertilizer efficiency was as low as 0.65, leaving potential room for improvement in fertilizer reduction. Based on research on chemical fertilizer application in the main wheat-producing regions across the country, Shi et al. [28] found that the efficiency of fertilizer use remained stable at around 45% from 1998 to 2013, with no significant improvement observed. Wang et al. [29] conducted calculations on the production efficiency of maize in 20 provinces of China from 2006 to 2016. They concluded that non-point source pollution is the primary factor leading to the decline in maize production efficiency. The study suggests the necessity of reducing the quantities of nitrogen and phosphorus fertilizers. Zhang et al. [30] also concluded the same conclusion in their research. They believed that unreasonable large amount of fertilizer application would not increase grain yield, but would reduce grain production efficiency. The fragmentation of cultivated land is the main reason for the low efficiency of fertilizer use [31]. The scale of cultivated land has a positive effect on improving the efficiency of fertilizer use [32, 33].

Based on the above literature, it is evident that reducing the quantity of chemical fertilizer application and improving the efficiency of fertilizer use are crucial measures for the high-quality development of agriculture. However, there are also some shortcomings. First, the focus of existing literature has often been concentrated on specific domains, such as the quantity of chemical fertilizer application or the efficiency of fertilizer use. There is a scarcity of literature that systematically examines and discusses the relationships among the quantity of chemical fertilizer application, the efficiency of fertilizer use, and grain production within the same framework. Second, there is scarce literature that explores the impact of reducing chemical fertilizer on grain production in the current context of fertilizer reduction. Additionally, few studies delve into the role of fertilizer use efficiency in the relationship between fertilizer application and grain production.

The possible marginal contributions of this paper lie mainly in the following: First, the main theoretical contribution of this paper is to elucidate the decision principles governing the quantity and efficiency of chemical fertilizer application in grain production. It offers a theoretical framework to explain the choice between increasing production through quantity or efficiency in the context of grain production. Second, the paper introduced the variable of fertilizer use efficiency into the analysis of the impact of fertilizer application on grain production. Fertilizer use efficiency is measured using the SBM model, and the study takes into account both expected and unexpected conditions of fertilizer use efficiency.

## 2. Theoretical analysis

### 2.1 Relationship between fertilizer inputs and food production

The following assumptions are given in this paper: First, farmers are assumed to be “rational individual” with homogeneous preferences, and they only care about maximizing food production in terms of factor inputs. Second, the factors in the agricultural production factor market are free to flow, and although farmers can allocate factors in agricultural production according to the profit of the product, they also face the constraints of factor flow and increased risk. Set the fixed resource possession of farmers engaged in food production as S. Farmers engaged in food production activities produce only two food crops, A and B, and the prices of the two food crops are the same, and the outputs are Q_1_ and Q_2_, respectively, and *q*_1_*q*_2_ is the production possibility frontier. Third, it is assumed that the main nutrients for food production come from fertilizers, where increasing the amount of fertilizer input will affect the food production of Crop A and increasing the efficiency of fertilizer use will affect the food production of Crop B. Farmers can voluntarily choose either of these ways to increase food production.

**Fig 1:**
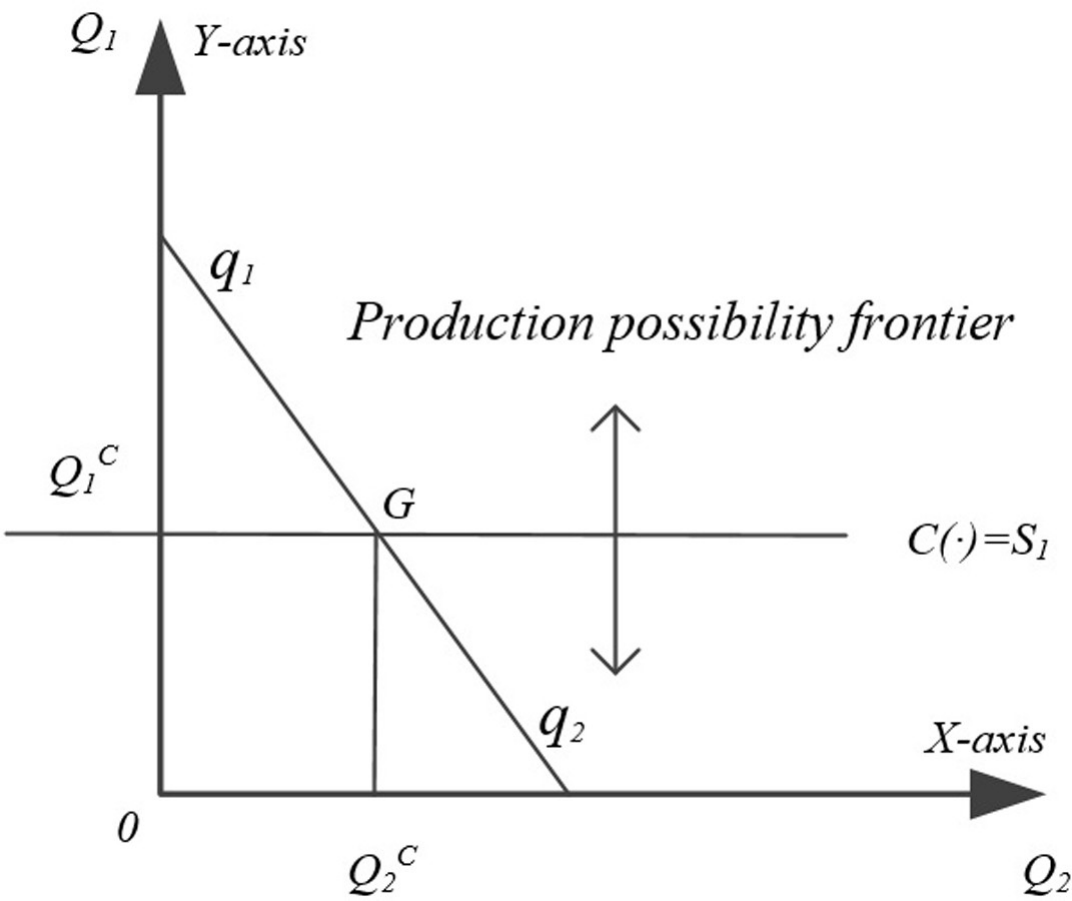
Potential impact of fertilizer reduction on household food production.

It should be noted fertilizer is the main input factor for both crops, and the capital used to purchase the fertilizer is fixed. Food crops in category A are affected by the amount of fertilizer applied, and reducing the amount of fertilizer applied to food crops in category A will lead to a reduction in food production; food crops in category B are affected by the efficiency of fertilizer use, and increasing the efficiency of fertilizer use in food crops in category B will increase food production, and reducing the amount of fertilizer applied to food crops in category A will increase food production. The capital savings from fertilizer application to food crops A are used to purchase more efficient fertilizers as a factor of production for food crops B. Changes in fertilizer application affect the factor production endowment such as soil fertility and risk level, so that food production is constrained, assuming that food crops in category B affected by fertilizer use efficiency are constrained by: C (·) = S1, S1 = C (F, E), where F is the fertilizer reduction for Crop A and E is the fertilizer use efficiency. Then the output of Crop B is Q_1_^C^, the output of food crops in Crop A The output of food crops is Q_2_^C^. The strategic interplay of constrained fertilizer application and efficiency of use causes the constraint condition C(·) to shift vertically, thereby influencing the equilibrium output of grains, as illustrated in Fig 1.

Based on the above potential effects of fertilizer reduction on grain yield, the paper summarizes the input mechanisms of fertilizer factors for different grain production. The fixed agricultural production resources are C(·) = C(F,E), F denotes the factor of production based on fertilizer dosage, E denotes the factor of production based on fertilizer efficiency, the initial level of farmers’ food production is C_0_, the initial agricultural output is Q_1_^0^, and when reducing the input of fertilizer factors, farmers change their factor input decisions according to the changes in the level of food production resources.

**Fig 2:**
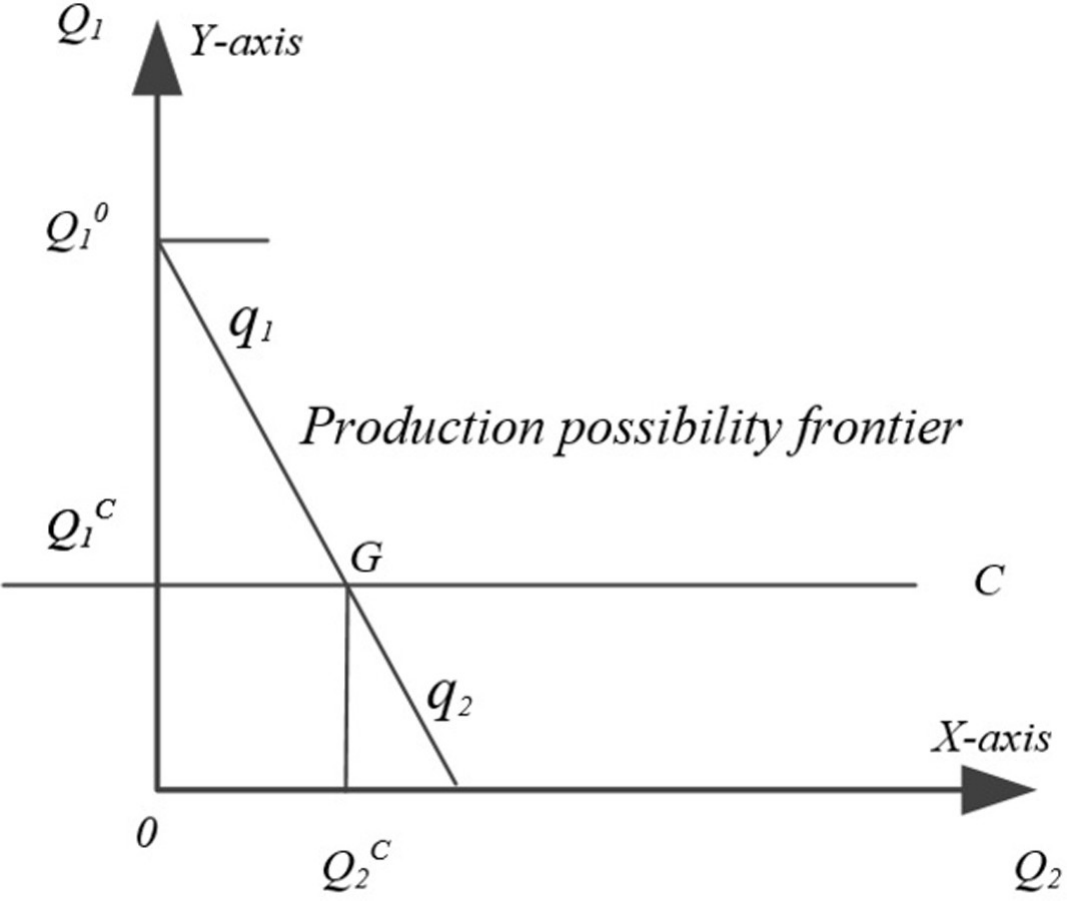
Fertilizer reduction and food production scenario 1.

Scenario 1: When the implementation of fertilizer reduction begins, if the yield reduction effect of fertilizer reduction |U(F)| is greater than the yield increase effect of fertilizer efficiency improvement |U€|, that is, |U(F)| €(E)|, it means that the increase in food production brought about by fertilizer efficiency improvement cannot compensate for the reduction in food production caused by fertilizer reduction, and the resource level of food production drops to become C limited by the constraints, the yield of Crop A food crops is Q_1_^C^, and there exists the reduced grain yield Q_1_^0^Q_1_^C^ brought by fertilizer application reduction is greater than the increased yield OQ_2_^C^ from fertilizer efficiency improvement, Q_1_^0^Q_1_^C^>OQ_2_^C^, at this time, farmers prefer to maintain the fertilizer application as much as possible or slowly reduce the fertilizer application to produce the production of Crop A, and meanwhile continue to improve the efficiency of fertilizer use to increase the total grain yield by increasing production of Crop B (Fig 2).

**Fig 3:**
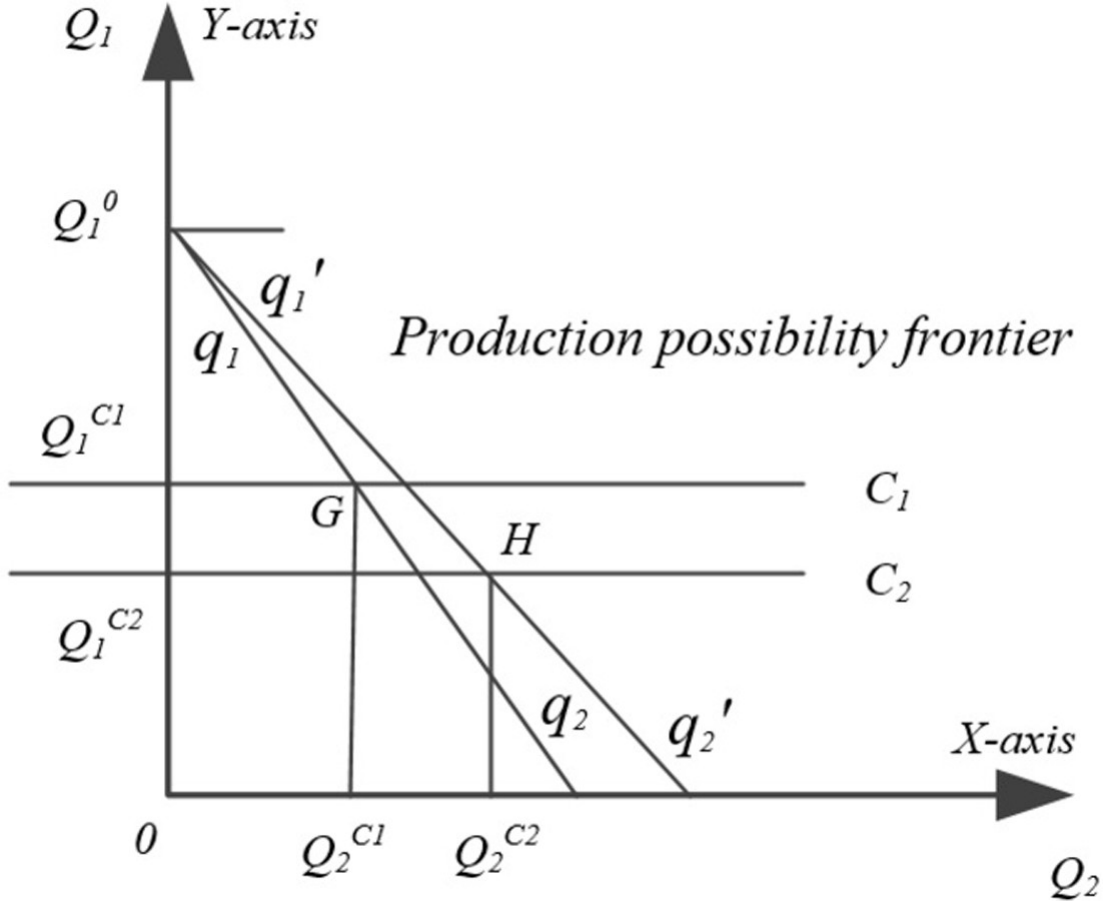
Fertilizer reduction and food production scenario 2 and scenario 3.

Scenario 2: As the fertilizer application reduction gradually increases, the excess factor capital from the fertilizer reduction becomes more and more sufficient for purchasing high-efficiency fertilizers or improving the efficiency of fertilizer use, and the yield-increasing effect of fertilizer efficiency improvement becomes gradually obvious. If point G is the equilibrium point between Crop A and Crop B, which means that the yield of Crop A is equal to that of Crop B under the factor combination at point G, that is, OQ_1_^C1^ = OQ_2_^C1^. At this time, the yield reduction effect of fertilizer reduction is equal to the yield increase effect of fertilizer efficiency improvement, that is, |U(F)| = |U(E)|. Then, the reduced grain yield from reduced fertilizer application by farmers is exactly offset by the increased yield from increased fertilizer efficiency, and under the strict constraint of fertilizer reduction, farmers will choose to increase the efficiency of fertilizer use for grain production (Fig 3).

Scenario 3: Continue to reduce the amount of fertilizer applied to Crop A, when the equilibrium point G between Crop A and Crop B is exceeded, the yield increase effect of fertilizer efficiency improvement exceeds the yield reduction effect of fertilizer reduction, that is, |U(F)| <|U(E)|, at this time, the resource level of food production decreases from C_1_ to become C_2_, the productivity of Crop B food crops is improved, and the production possibility boundary is shifted from *q*_1_*q*_2_ line shifted right to *q*_1_’*q*_2_’, and the reduced grain yield Q_1_^C1^Q_1_^C2^ brought by fertilizer reduction is smaller than the increased grain yield Q_2_^C1^Q_2_^C2^ brought by fertilizer efficiency improvement, and the point H is the output combination of Crop A and Crop B. Then, in order to increase grain yield, farmers will make the decision to increase the efficiency of fertilizer use. Especially when the yield-increasing effect of chemical fertilizer efficiency improvement is far greater than the yield-reducing effect of fertilizer reduction, the yield of Crop B obtained by improving the efficiency of chemical fertilizer use exceeds the initial yield Q_1_^0^ of Crop A. At this time, farmers will significantly reduce the amount of fertilizer applied to Crop A, which will then be transformed into efficient fertilizer input to the production of Crop B (Fig 3).

### 2.2 Determination of criteria for excessive application of fertilizers

**Fig 4:**
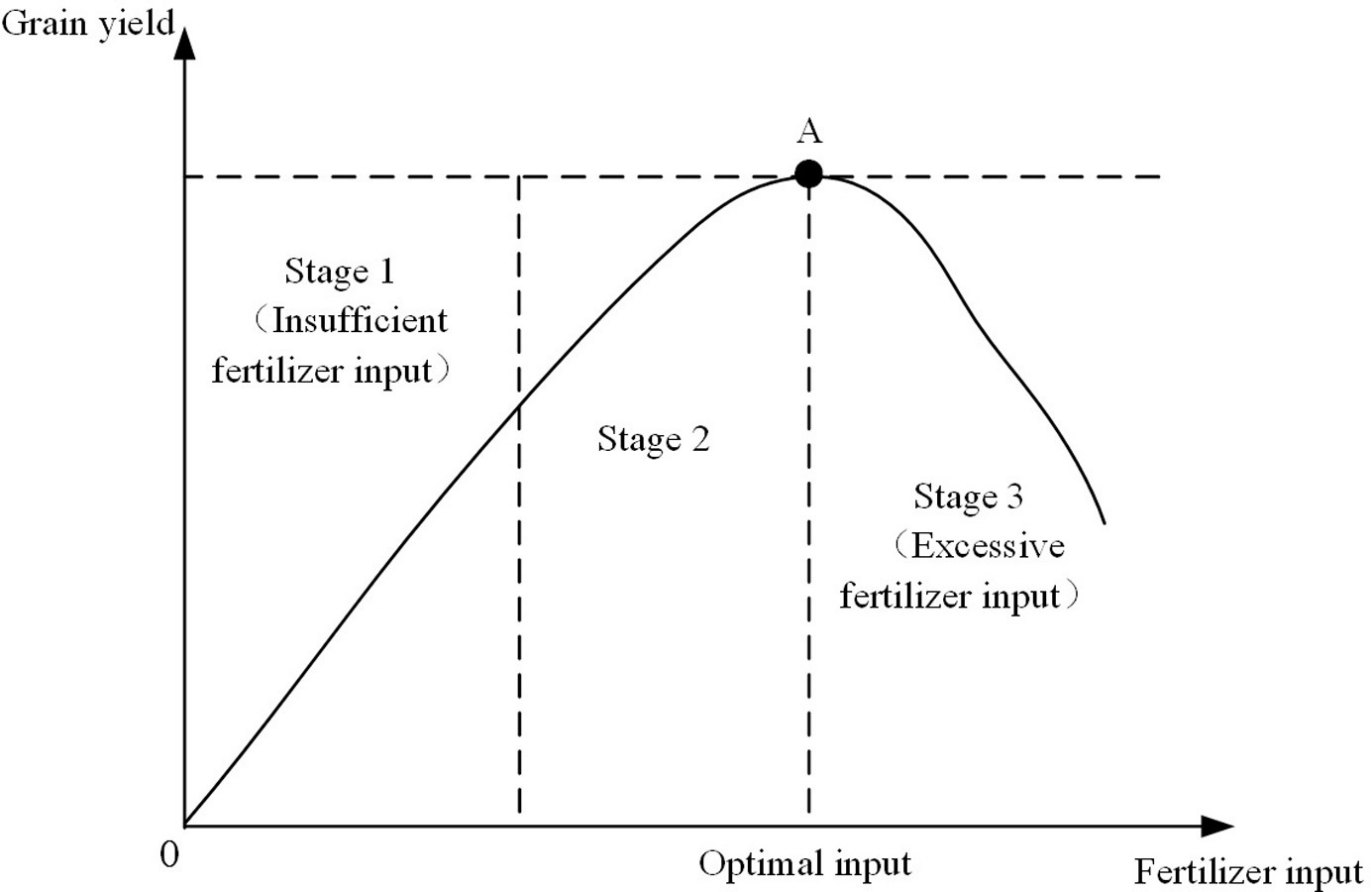
Schematic diagram illustrating fertilizer application standards.

Fig 4 is a schematic diagram for assessing whether fertilizer is being used excessively. In grain production, the principle is not that more fertilizer input is always better. It typically follows the law of diminishing marginal returns, where the optimal threshold of fertilizer input is determined by maximizing grain yield. Fig 4 depicts that, in the stage 1, the application of fertilizers exerts a pronounced promotional impact on grain yield. Concurrently, with the continuous escalation of grain yield, the promotional effect of fertilizer input on grain yield gradually diminishes. Upon entering the stage 2, the incremental effect of increasing fertilizer input on grain yield gradually diminishes to zero. When the promotional effect of fertilizer input on grain yield becomes zero (point A), the fertilizer input has reached the optimal threshold. This indicates that, at the point A of fertilizer input, grain yield can reach its maximum value. If the fertilizer input continues to increase, it will enter the stage 3. At this point, not only does the fertilizer input fail to promote an increase in grain yield, but it leads to a gradual decrease in grain yield. Therefore, during the first and second phases, increasing fertilizer input can promote an increase in grain yield, reaching the optimal fertilizer input at point A. Subsequently, if the fertilizer input continues to increase, it will result in a decrease in grain yield.

## 3. Relationship between fertilizer reduction and grain yield

### 3.1 Tapio decoupling model

"Decoupling" comes from the technical term of physics and is applied to resource and environmental economics, indicating a state in which economic development and environmental pollution coexist and show different trends [34]. The Tapio decoupling model can clearly show the trade-offs between variables and effectively avoid the problem of bias due to the instability of outliers [35]. In this paper, the Tapio decoupling model is used to construct a dynamic relationship between fertilizer application and grain yield, which is "decreasing with increasing" or "increasing and decreasing at the same time", and to characterize the trend of fertilizer changes in the process of stable and increasing grain yield in China.

$$E_{i}=\frac{\Delta F/F}{\Delta G/G}=\frac{(F_{i+1}-F_{i})/F_{i}}{(G_{i+1}-G_{i})/G_{i}}$$ (1)

where, *E*_*i*_ denotes the decoupling elasticity coefficient in year *i*, *F* denotes the total fertilizer input, *G* denotes the total grain production, and *i* represents the year *i*.

The types of decoupling can be classified into eight categories according to the elasticity value: strong decoupling, weak decoupling, declining decoupling, growing connection, declining connection, growing negative decoupling, weak negative decoupling, and strong negative decoupling (Table 1).

**Table 1:** Decoupling scale of grain yield increase and fertilizer consumption.

Decoupled state	discriminant condition			meaning
	*△G/G*	*△F/F*	*E*	
Strong decoupling	>0	<0	E<0	Grain yield increased with decreased fertilizer application
Weak decoupling	>0	>0	0<E<0.8	Grain yield increased with a gradual growth in fertilizer application
Declining decoupling	<0	<0	E>1.2	Grain yield slow declined with large declines in fertilizer application
Growth Connection	>0	>0	0.8<E<1.2	Fertilizer application increased in tandem with grain yield
Declining decoupling	<0	<0	0.8<E<1.2	Fertilizer application declined in tandem with declining grain yield
Negative Growth Decoupling	>0	>0	E>1.2	Grain yield slow increased with significant increase in fertilizer application.
Weak Negative Decoupling	<0	<0	0<E<0.8	Grain yield declined with a slow decline in fertilizer application
Strong negative decoupling	<0	>0	E<0	Grain yield declined with increased fertilizer application

### 3.2 Decoupling analysis of fertilizer application and grain yield

The results in Table 2 show that from 2004 to 2014, the amount of fertilizer applied in China was always in a state of growth, same as the grain production, indicating that fertilizer has an important role in increasing grain production. From 2015 to 2019, the amount of fertilizer applied and grain production were in an alternating relationship of declining decoupling and strong decoupling, and the amount of fertilizer applied had a significant decline, while grain production was in a trend of increasing and decreasing, and Grain production was basically maintained at about 650 million tons.

**Table 2:** Decoupling status of fertilizer application and grain yield from 2004 to 2019.

year	Grain production (million tons)	Total fertilizer (million tons)	△G/G	△F/F	EF	Decoupling Status
2004	46946.95	4636.6	0.03	0.03	0.90	Growing Connections
2005	48402.20	4766.2	0.03	0.03	1.17	Growth Connection
2006	49804.20	4927.7	0.01	0.04	2.99	Growth Negative Decoupling
2007	50413.90	5107.8	0.06	0.03	0.43	Weak decoupling
2008	53434.30	5239	0.01	0.03	3.33	Growth Negative Decoupling
2009	53940.90	5404.4	0.04	0.03	0.80	Weak decoupling
2010	55911.30	5561.7	0.05	0.03	0.49	Weak decoupling
2011	58849.30	5704.2	0.04	0.02	0.59	Weak decoupling
2012	61222.60	5838.8	0.03	0.01	0.42	Weak decoupling
2013	63048.20	5911.9	0.01	0.01	0.98	Growth connected
2014	63964.80	5995.9	0.03	0.010	0.14	Weak decoupling
2015	66060.30	6022.6	-0.01	-0.01	25.14	Decline decoupling
2016	66043.50	5984.1	0.01	-0.02	-11.74	Strong decoupling
2017	66160.70	5859.4	-0.01	-0.04	6.26	Decline Decoupling
2018	65789.20	5653.4	0.01	-0.04	-4.89	Strong decoupling
2019	66384.3	5403.6	0.01	-0.05	-5.16	Strong decoupling

Note: Data from “China Statistical Yearbook”, “China Environmental Statistical Yearbook”

Specifically, from 2004 to 2005, fertilizer application and grain yield were in a decoupling relationship, and fertilizer application and grain yield increased simultaneously, indicating that the marginal utility of fertilizer in grain production was relatively large at this time, and fertilizer input could bring about a certain percentage of grain yield increase; in 2006, fertilizer application and grain yield were in a negative decoupling relationship, and fertilizer application increased significantly while grain yield grew slowly. In 2006, the marginal utility of fertilizer in grain production gradually declined, and the large amount of fertilizer input could not bring the expected increase in grain yield, but would increase the production cost; in 2007, the amount of fertilizer application was in a weak decoupling relationship with grain yield, the amount of fertilizer application increased slowly, and grain yield kept growing, at this time, fertilizer still had a certain marginal utility, and appropriate fertilizer input was beneficial to the increase in grain yield; in 2008, the amount of fertilizer application was in a weak decoupling relationship with grain yield, and the amount of fertilizer application increased slowly, and grain yield kept growing. fertilizer application and grain yield are in a negative decoupling relationship, fertilizer application decreases significantly and grain yield grows slowly, at this time, fertilizer application decreases, but its marginal utility on grain yield increase shows positive; from 2009 to 2012, fertilizer application and grain yield are in a weak decoupling relationship, fertilizer application grows slowly and grain yield grows, at this time, the marginal utility of fertilizer application on grain yield is in a gradually increasing state The marginal utility of fertilizer application on grain yield was in a gradually increasing state, with the increase of fertilizer application, grain yield was also in an increasing state; in 2013, fertilizer application and grain yield were in a growing connection decoupling relationship, fertilizer application and grain yield grew simultaneously, at this time, the marginal utility of fertilizer application on grain yield was larger, and the two were in a better balance; in 2014, fertilizer application and grain yield were in a weak decoupling relationship, fertilizer application grew slowly and grain yield increased, at this time, the marginal utility of fertilizer From 2015 to 2019, fertilizer application and grain yield were in an alternating relationship of declining decoupling and strong decoupling, and in 2016, fertilizer application showed the first decline since 2004, accompanied by the first decline in grain yield. It shows that the Action Plan implemented in 2015 had a significant effect on fertilizer reduction and was an important reason for the first decline in fertilizer application, and also shows that China’s grain production has long relied on fertilizer inputs and that fertilizer plays an important role in grain production. It is worth paying attention to the fact that although the fertilizer application has been in a state of decline or significant decline during this period, grain production has been in a state of increase or slow decline, and the magnitude of change in fertilizer use is obviously greater than the magnitude of change in grain production, which also shows from the side that the marginal utility of fertilizer on grain is gradually decreasing, indicating that grain production can also increase under the condition of fertilizer reduction, and in a certain Under the premise of degree, You can have your cake and eat it too.

Overall, the decoupling relationship between fertilizer application and grain yield in different years shows that fertilizer, as an indispensable production material for grain production, plays an important role in grain production and is an indispensable production factor for grain production, but it is also noted that fertilizer application is still subject to the law of diminishing marginal returns, and appropriate fertilizer input is necessary, although excessive Although excessive fertilizer input is beneficial to short-term food production, the problem of agricultural surface pollution brought about by it is a potential "hidden danger" that cannot be ignored in future food production. Therefore, in the case of excessive fertilizer application, reduced fertilizer inputs can only have a positive effect on food production.

## 4. Empirical test of the effect of fertilizer reduction on grain yield

### 4.1 Model Setting

Due to the diverse situations faced by different provinces, there may be omitted variables that do not vary over time. Therefore, considering the use of a two-way fixed effects model. The paper uses the software stata 17.0 to derive data as balanced panel data, with a structure of n>T, so the data are short panel data. Since different provinces face different situations and there may be omitted variables that do not change over time, we use the fixed effects model. Meanwhile, the Hausman test was conducted on the panel data, and the results showed that P<1%, strongly rejecting the original hypothesis, thus fixed-effects model should be used. Referring to Kirwan (2009) [36] and Bustos et al. (2016) [37], the following analytical model was constructed:

$$\text{ln}Grain_{ij}=\alpha +\beta_{1}Fer_{ij}+\beta_{1}Eff_{ij}+\lambda X_{ij}+\mu_{i}+\varphi_{j}+\delta_{ij}$$ (2)

In Eq (2), i denotes province, j denotes year, the explanatory variable *Grain* is total grain production, *Fer* is fertilizer application, *Eff* is fertilizer use efficiency, and *X*_*ij*_ is a set of control variables affecting grain production, *μ*_*i*_ denotes provincial control variables, which mainly include variables affecting grain production such as regional characteristics, rural labor, and crop characteristics, *φ*_*i*_ denotes time control variables related to grain production, and *δ*_*ij*_ is a residual term.

The model of the basic expression after the introduction of the cross term model is:

$$\text{ln}Grain_{ij}=\alpha +\beta_{1}Fer_{ij}+\beta_{2}Fer_{ij}\times Var_{ij}+\lambda X_{ij}+\mu_{i}+\varphi_{j}+\delta_{ij}$$ (3)

In Eq (3), *Var*_*ij*_ denotes the fertilizer application efficiency and the dependent variable is estimated in Eq (2) separately, *Fer*_*ij*_×*Var*_*ij*_ denotes the interaction term, denotes the interaction term between fertilizer application and fertilizer use efficiency separately, and the meaning and parameters of the remaining variables are consistent with Eq (2).

### 4.2 Variable settings and data description

#### 4.2.1 Dependent variable

The paper treats total grain yield as the explanatory variable, and to avoid heteroskedasticity bias, the dependent variable total grain yield is treated logarithmically.

#### 4.2.2 Main independent variable

The main independent variable is fertilizer application amount (discounted amount). Fertilizer application has always been concerned by scholars, and many scholars use it as an important indicator for studying food production and agricultural pollution. At the same time, the *Action Plan* also puts forward the task of zero growth in fertilizer use, so fertilizer application is selected as the main independent variable in the article, and the key explanatory variable, fertilizer application, is logarithmically treated in order to keep the data smooth and reduce data differences. To test the robustness of the estimation results, fertilizer application intensity was used as a replacement variable for fertilizer application in this paper, and fertilizer application was measured against the total area sown to crops.

#### 4.2.3 Control variables

The control variables of the article are set in four aspects: regional variables, rural labor variables, food crop characteristics, and natural characteristics.

1. Regional Characteristics. ① Regional Gross Domestic Product (*GDP*), indicating the overall economic development level of the region; ② Effective Irrigated Area (*irrigation*), reflecting the agricultural production capacity of the region; ③ Disaster Resilience (*DR*), calculated as (Affected Area—Crop Failure Area) / Affected Area, characterizing the risk resistance capability in agricultural production.
2. Labor-related Variables. ① Agricultural and forestry employment (*employee*). Represents the labor force engaged in agricultural production activities. For the missing data in the number of people engaged in agricultural and forestry activities, cubic spline interpolation is applied for completion.② The disposable income of rural residents (*income*), which indicates the income level of rural residents. ③ Grain production price index of the previous year (*previous price*). Used to represent the lagged effect of grain prices on grain production.
3. Policy Variables (*policy*). The article used the "Action Plan for Zero Growth in Fertilizer Use by 2020" issued by the original Ministry of Agriculture of China in 2015 as a time point for policy intervention. A policy dummy variable is set, with years before 2015 assigned as 0 and years after 2015 assigned as 1.
4. Natural Feature Variables. The article uses the annual average temperature (*temperature*) and annual precipitation (*precipitation*) of each province in China to represent the region’s precipitation and climate characteristics. Additionally, virtual terrain feature variables (*terrain*) are set as shown in **Table 3**. The terrain classifications for each province are obtained from the "China County (City) Social and Economic Statistical Yearbook (2012)." Plain provinces are assigned a value of 1, while hilly provinces are assigned a value of 0.

**Table 3:** Topographic classification of Chinese provinces.

Terrain classification	Province	Share of hilly and mountainous districts	Terrain classification	Province	Share of hilly and mountainous districts
Hilly area	Guangxi	0.92	Plain area	Hainan	0.579
	Guizhou	0.878		Zhejiang	0.569
	Tibet	0.857		Heilongjiang	0.566
	Yunnan	0.848		Jilin	0.521
	Qinghai	0.822		Shannxi	0.478
	Fujian	0.803		Liaoning	0.397
	Sichuan	0.755		Ningxia	0.389
	Hunan	0.703		Anhui	0.385
	Inner Mongolia	0.696		Shandong	0.373
	Gansu	0.687		Henan	0.339
	Chongqing	0.684		Hebei	0.288
	Jiangxi	0.674		Jiangsu	0.091
	Hubei	0.671		Xinjiang	0.085
	Hubei	0.641		Beijing	0.063
	Shanxi	0.607		Tianjin	0.063
	-	-		Shanghai	0.000

Data source: Compiled based on the "China County (City) Social and Economic Statistical Yearbook (2012)". (2012 data was chosen because this database has been using the county-level terrain classification from 2013 onwards, directly continuing the classification used before 2012, without separately listing the names of mountainous and hilly counties).

To reduce bias caused by heteroscedasticity, the article applies logarithmic transformation to the variables: regional GDP, labor force in agriculture, forestry, animal husbandry, and fishery, effective irrigated area, rural residents’ disposable income, the previous year’s cereal price index, and annual precipitation.

#### 4.2.4 Mechanistic variables

There is no doubt that fertilizer plays a key role in food production, but also due to the low efficiency of fertilizer use leads to problems such as surface pollution [38], and improving the efficiency of fertilizer use becomes a "two-pronged strategy" to promote stable grain production and increase yield while reducing agricultural surface pollution. Therefore, the paper considers fertilizer use efficiency as an important indicator of grain production under the constraints of fertilizer reduction. The stochastic frontier production function (SFA) and data envelopment analysis (DEA) are the main methods to measure the efficiency of fertilizer use. The shortcomings of SFA are that it ignores the constraints of resources and environment, which cannot fully reflect the connotation of green and ecological livability and is not applicable to the measurement of fertilizer efficiency under the constraints; the shortcomings of CCR and BCC models under DEA framework are that they fail to take into account the non-desired outputs and cannot comprehensively consider the relationship between the slack input-output of fertilizer and agricultural surface pollution. For this reason, Tone [39] proposed the SBM model, which incorporates the slack input-output of fertilizer into the measurement of fertilizer efficiency, effectively solving the problem of measuring fertilizer efficiency under environmental constraints with the expression:

$$\begin{array}{l} \rho^{*}=\min\rho =\frac{1-\frac{1}{m}\sum_{i=1}^{m}\frac{s_{i}^{-}}{x_{ij}}}{1+\frac{1}{s+k}(\frac{\sum_{r=1}^{s}s_{r}^{+}}{y_{rj}}+\sum_{t=1}^{k}\frac{s_{t}^{-}}{z_{tj}})}\\ s.t.\left\{\begin{array}{cc} \sum_{j=1}^{n}\lambda_{j}x_{ij}+s_{i}^{-}=x_{ij,} & i=1,\cdots ,m\\ \sum_{j=1}^{n}\lambda_{j}x_{ij}-s_{r}^{+}=y_{rj}, & r=1,\cdots ,s\\ \sum_{j=1}^{n}\lambda_{j}z_{tj}+s_{t}^{-}=z_{tj}, & t=1,\cdots ,k\\ \lambda_{\text{j}}\geq 0,s_{i}^{-}\geq 0,s_{\text{r}}^{+}\geq 0, & i=1,\cdots ,m \end{array}\right. \end{array}$$ (4)

In Eq (4), p* denotes fertilizer input slack, *x*_*j*_ is the input vector, *x*_*ij*_ denotes the input of the i-th factor, $s_{i}^{-},s_{r}^{+},s_{t}^{-}$denotes the slack variables of fertilizer input, desired output and undesired output, respectively, *x*_*ij*_,*y*_*rj*_,*z*_*tj*_ denotes the input factor, desired output and undesired output of j decision units *DMU*_*j*_, respectively; *m*,*s*,*k* denotes the quantity of input factor, desired output and undesired output, respectively, *λ* is the weight of input factor, desired output and undesired output.

In this paper, labor, fertilizer, agricultural film, pesticide, energy and agricultural water were selected as factor input indicators, total agricultural output value was taken as expected output, and agricultural carbon emission was taken as non-expected output [40]. The calculation formula of fertilizer efficiency was as follows:

$$FE_{j}^{i}=\frac{F_{j}^{i}-S_{jF}^{i}}{F_{j}^{i}}$$ (5)

In Eq (5), $FE_{j}^{i},F_{j}^{i},S_{jF}^{i}$ is the fertilizer use efficiency, the actual fertilizer input and the fertilizer input slack for *j* decision units in period *t*, respectively.

#### 4.2.5 Selection of tool variables

The amount of fertilizer application is mainly influenced by the subjective factors of farmers, the main agricultural producers, and the difference in cognitive level is the key to how much fertilizer is applied [41], and the individual farmers’ literacy level and their knowledge of the hazards of fertilizer significantly affect the overall cognitive level of farmers, and this cognitive level has further influence on the scientific fertilizer application behavior of farmers [42], that is, the amount of fertilizer application has endogeneity problem. Ge and Zhou [43] argue that fertilizer price control and subsidy policies have led to distortions in factor markets that may stimulate an increase in fertilizer inputs. For this reason, the production price index of chemical fertilizer (based on 2004) is used to reflect the price changes of chemical fertilizer, and the production price index of chemical fertilizer is used as the instrumental variable of fertilizer application [44].

Scholars argue that because farmers lack systematic knowledge of fertilizer application, they tend to base their agricultural production on their personal production experience, and at the same time, farmers, as "risk-averse" investors, usually fall into the misconception that the higher the fertilizer application, the higher the grain yield [45]. Wu and Ge (2019). suggested that farmers’ education level affects their fertilizer application behavior, because farmers with higher education level are more receptive to new technologies, tend to choose scientific fertilizer application methods, adopt more organic fertilizers, and reduce the amount of chemical fertilizers applied [46].

Therefore, per capita education level was selected as the instrumental variable of fertilizer application, and 0, 6, 9, 12, 12, 15, 16 and 19 years of education were assigned to the 8 levels of unattended school, primary school, junior high school, senior high school, secondary vocational school, junior college, undergraduate, postgraduate and above [47]. The educational level of peasant households is expressed by the per capita educational years of agricultural labor population in each province:

$$h=\frac{\sum e_{i}\times P_{i}}{T_{p}}$$ (6)

In Eq (6), *e*_*i*_ denotes the number of years of schooling for the ith level of education, *P*_*i*_ denotes the number of people with i years of education in the rural population, and *T*_*p*_ denotes the total population of secondary school age or older in the rural population.

Table 4 reports the results of descriptive statistics for each variable. The data in this paper mainly comes from China Statistical Yearbook, China Rural Statistical Yearbook, China Education Statistical Yearbook, and other statistical yearbooks. Statistical yearbooks typically compile various official statistics, including but not limited to agricultural production, population statistics, etc. These data are generally obtained through government agencies, organizations, or experimental studies. Therefore, the data in this paper can be categorized as general farm-level survey data.

**Table 4:** Descriptive statistical analysis of variables.

Variable	Name	Max	Min	Mean	SD	Obs	Data source
Total grain production (million tons)	*ln_grain*	8.923	3.360	7.023	1.230	485	China Statistical Yearbook
Fertilizer application amount (million tons)	*ln_fer*	6.574	1.400	4.740	1.181	485	China Rural Statistical Yearbook
Fertilizer application efficiency (%)	*eff*	1	0.095	0.398	0.245	485	Calculated from
Disaster Resilience (%)	*DR*	106	1	59.400	20.720	485	China Rural Statistical Yearbook
Gross regional product (billion yuan)	*ln_GDP*	11.587	5.350	9.249	1.146	485	China Statistical Yearbook
Effective irrigated area (thousand hectares)	*ln_irrigation*	8.720	3.040	6.937	1.314	485	China Statistical Yearbook
Number of people employed in agriculture, forestry, animal husbandry and fishery (10,000 people)	*ln_employee*	8.090	3.089	6.369	1.084	485	National Bureau of Statistics
Disposable income of rural residents (yuan/person)	*ln_income*	11.041	7.451	9.082	0.848	485	China Rural Statistical Yearbook
Cereal production price index of the previous year (%)	*previous price*	141.530	79.900	105.151	8.438	469	China Agricultural Price Survey Yearbook
Average annual temperature (°C)	*tem*	23.6	3.956	13.573	4.696	485	National Meteorological Science Data Center
Average annual precipitation (mm)	*ln_precipitation*	10	7.600	9.023	0.504	485	National Meteorological Science Data Center
Terrain (1 = plain area, 0 = hilly area)	*terrain*	1	0.000	0.505	0.500	485	China Statistical Yearbook
Whether the fertilizer reduction policy is implemented (1 = yes, 0 = no)	*policy*	1	0.000	0.311	0.464	485	State Council (PRC)
Years of education per capita (years)	*edu*	12.686	3.738	8.589	1.191	485	China Education Statistical Yearbook
Fertilizer production price index (%)	*price*	145.764	80.954	104.271	9.628	432	China Agricultural Products Price Survey Yearbook
Rice Yield (10000 tons)	*Rice*	2819.30	0.000	675.162	740.968	469	China Rural Statistical Yearbook
Wheat Yield (10000 tons)	*Wheat*	3741.80	0.000	403.686	732.06	468	China Rural Statistical Yearbook
Corn Yield (10000 tons)	*Corn*	3982.20	0.800	652.151	805.281	478	China Rural Statistical Yearbook
Nitrogen Fertilizer (10000 tons)	*Nitrogen Fertilizer*	245.500	1.500	75.028	55.668	485	China Rural Statistical Yearbook
Phosphorus Fertilizer (10000 tons)	*Phosphorus Fertilizer*	121.700	0.300	25.982	23.644	485	China Rural Statistical Yearbook
Potash fertilizer (10000 tons)	*Phosphorus Fertilizer*	64.600	0.100	19.040	16.261	485	China Rural Statistical Yearbook
Organic fertilizer (10000 tons)	*Organic fertilizer*	337.300	1.000	61.074	58.167	485	China Rural Statistical Yearbook

Note: Organized by the author.

### 4.3 Estimation results and analysis

#### 4.3.1 Examination of instrumental variables

The paper introduced the fertilizer production price index and per capita education level as instrumental variables into the model and conducts over-identification tests, weak instrument tests, and DWH tests for each instrumental variable (Table 5). The results of the over-identification test show that the p-values for each instrumental variable in Estimates 1 to 4 are 0.2057, 0.2131, 0.7094, and 0.7567, respectively, accepting the null hypothesis that "all instrumental variables are exogenous." The results of the weak instrument test indicate that the F-values for each instrumental variable are 162.391, 158.939, 43.0949, and 43.1411, all exceeding 10, indicating the absence of weak instrumental variables. To further verify the existence of weak instrumental variables, the empirical test was conducted using the limited information maximum likelihood method, and the results still indicated the absence of weak instrumental variables. The results of the heteroskedasticity robust DWH test showed that all instrumental variables were significant at the 1% level, so fertilizer application was considered as an endogenous variable. Comparing the IV estimation with the ordinary fixed effects model, it can be observed that the estimated coefficients of fertilizer application change significantly with the inclusion of instrumental variables, indicating that the omitted endogenous variables do cause the problem of biased results.

**Table 5:** Analysis of basic regression results.

	Estimate 1		Estimate 2		Estimate 3		Estimate 4	
	FE	2SLS	FE	2SLS	FE	2SLS	FE	2SLS
*ln_fer*	0.720***	0.8334***	0.7127***	0.8271***	0.7833***	0.8622***	0.7806***	0.8627***
	(-0.258)	(-0.021)	(-0.243)	(-0.02)	(-0.253)	(-0.076)	(-0.231)	(-0.076)
*eff*			-0.1171	0.3451**			-0.1484	-0.0605
			(-0.177)	(-0.159)			(-0.171)	(-0.057)
*ln_GDP*					-0.2837***	-0.2979***	-0.2711**	-0.2880***
					(-0.094)	(-0.064)	(-0.103)	(-0.062)
*DR*					-0.0010***	-0.0015**	-0.0011***	-0.0016**
					(0.00)	(-0.001)	(0.00)	(-0.001)
*ln_irrigation*					-0.0167	0.1411***	-0.0191	0.1429***
					(-0.02)	(-0.042)	(-0.022)	(-0.041)
*ln_employee*					0.0331	0.4090***	0.0369	0.3979***
					(-0.053)	(-0.059)	(-0.057)	(-0.058)
*ln_income*					-0.2595	0.6341***	-0.3089	0.6073***
					(-0.2)	(-0.133)	(-0.224)	(-0.129)
*previous price*					-0.0007	-0.0041*	-0.0004	-0.0040*
					(-0.001)	(-0.002)	(-0.001)	(-0.002)
*tem*					0.0069	-0.1162***	0.003	-0.1173***
					(-0.027)	(-0.004)	(-0.03)	(-0.004)
*ln_precipitation*					0.1071*	0.5655***	0.1080*	0.5768***
					(-0.056)	(-0.056)	(-0.054)	(-0.057)
*terrain*					-0.092	-0.0711**	-0.092	-0.0688**
					(0.100)	(-0.031)	(0.095)	(-0.031)
*policy*					0.9788***	0.0746	1.1161***	0.1393
					(-0.322)	(-0.254)	(-0.396)	(-0.258)
Year fixed effect	YES	YES	YES	YES	YES	YES	YES	YES
Province fixed effect	YES	YES	YES	YES	YES	YES	YES	YES
Constant	3.582***	3.133***	3.6420***	3.085***	6.6914***	-6.2989***	7.0353***	-6.1883***
	(-1.187)	(-0.114)	(-1.109)	(-0.117)	(-1.433)	(-0.745)	(-1.513)	(-0.721)
Observations	485	432	485	432	469	416	469	416
R-squared	0.444	0.843	0.45	0.845	0.514	0.952	0.524	0.952
Over-identification test		0.206		0.2131		0.7094		0.7567
Weak instrumental variable F-value		162.391		158.939		43.0949		43.1411
DWH test		<1%		<1%		<1%		<1%

Note: ***, ** and * indicate significant at the 1%, 5% and 10% levels, respectively; standard errors of clustering are in parentheses (same below).

#### 4.3.2 Analysis of model estimation results

Estimates 1 to 4 report the effects of the main independent variables fertilizer application, fertilizer use efficiency and series of control variables on grain yield (Table 5). Among them, estimation 1 is the regression result of the model without the inclusion of any control variables; estimation 2 is the regression result of the model with the inclusion of control variables; and estimation 3 is the regression result of the model with the inclusion of fertilizer use efficiency and all control variables. Overall, the model results indicate that fertilizer application significantly and positively affects grain yield at the 1% level with or without the inclusion of control variables or with or without the inclusion of fertilizer use efficiency, indicating that fertilizer still plays an important role in grain production that is difficult to replace and that an increase in fertilizer application still contributes to higher grain yield. However, with the inclusion of control variables, the effect of fertilizer application on grain yield increase gradually weakened. The results of the estimation 1 model showed that when no control variables were included, fertilizer application positively affected grain yield at the 1% significance level, with grain yield improving by 0.8334 units for every 1 unit increase in fertilizer application.

Estimate 2 results show that when fertilizer use efficiency is included, fertilizer application significantly and positively influences grain production at the 1% significance level. For each additional unit of fertilizer application, grain production increases by 0.8271 units. Compared to the model results without including fertilizer use efficiency, the enhancing effect of fertilizer application on grain production decreases by 0.63 percentage points. This might be attributed to the long-term excessive use of large amounts of fertilizer, which, while beneficial for the growth of cereal crops, can lead to a decline in land quality, agricultural non-point source pollution, and issues such as soil compaction and acidification. Excessive fertilizer use hampers the full effectiveness of fertilizer, creating a vicious cycle of "increasing fertilizer input—grain yield increase—increasing fertilizer input." This not only results in agricultural non-point source pollution but also increases the dependence of grain production on fertilizers. On the other hand, fertilizer use efficiency also significantly and positively influences grain production at the 5% significance level. For each unit increase in fertilizer use efficiency, grain production increases by 0.3451 units, indicating that fertilizer use efficiency, in conjunction with fertilizer application, forms a combined force for grain yield increase.

Estimate 3 results indicate that when control variables are included without incorporating fertilizer use efficiency, fertilizer application significantly and positively influences grain production at the 1% significance level. For each additional unit of fertilizer application, grain production increases by 0.8622 units, suggesting that the enhancement of agricultural production capacity, such as economic development level and disaster resistance, is conducive to increasing grain output.

Estimate 4 model results show that when fertilizer use efficiency and all control variables are included, fertilizer application still significantly and positively influences grain production at the 1% significance level. For each additional unit of fertilizer application, grain production increases by 0.8627 units. Compared to Estimate 1 model results, the enhancing effect of fertilizer application on grain production increases by 2.9 percentage points. Moreover, with the inclusion of control variables, fertilizer use efficiency does not significantly affect grain production. This indicates that fertilizer use efficiency in China still needs improvement to reduce the dependency of grain production on fertilizer application.

#### 4.3.3 Mechanism analysis of grain increase under the constraint of fertilizer reduction

Table 6 reports the regression results after introducing the interaction term between "fertilizer application" and "fertilizer use efficiency" into the model. Estimate (1) is the regression result without incorporating instrumental variables, and Estimate (2) is the regression result with instrumental variables. The p-value of the overidentification test in Estimate (2) is 0.8643, indicating that instrumental variables are exogenous. The F-value in the weak instrument test is 19.1731, greater than 10, suggesting the absence of weak instrumental variables. The heteroscedasticity-robust DWH test results show that all instrumental variables are significant at the 1% level, indicating that fertilizer application is an endogenous variable.

**Table 6:** Mechanism analysis of grain increase under the constraint of fertilizer reduction.

	(1)	(2)
	FE	2SLS
*ln_fer×eff*	0.2977***	0.2805*
	(-0.028)	(-0.151)
*ln_fer*	0.3270***	0.6744***
	(-0.064)	(-0.162)
*eff*	-1.5363***	-1.5069*
	(-0.14)	(-0.785)
Control variables	Control	Control
Constant	5.0606***	-5.6615***
	-1.002	-0.816
Observations	469	416
Year fixed effect	YES	YES
Province fixed effect	YES	YES
R-squared	0.624	0.956
Over-identification test		0.8643
Weak instrumental variable F-value		19.1731
DWH test		<1%

Note: ***, **, and * respectively indicate significance at the 1%, 5%, and 10% levels; standard errors in parentheses.

The results of Estimate (2) in Table 6 show that fertilizer application significantly and positively promotes an increase in grain production at the 1% level. The interaction term between fertilizer application and fertilizer use efficiency also significantly and positively influences grain production at the 10% level, indicating that fertilizer use efficiency enhances the promoting effect of fertilizer application on grain production, exhibiting a significant positive regulatory effect. When fertilizer use efficiency increases by 1 unit, the promoting effect of fertilizer application on grain production will increase by 0.2805 units. At the same time, fertilizer use efficiency negatively affects grain production at the 1% level, opposite to the direction of the impact of fertilizer application on grain production. This suggests a clear substitution relationship between fertilizer use efficiency and fertilizer application, meaning that reducing fertilizer application and increasing fertilizer use efficiency can still promote an increase in grain production.

#### 4.3.4 Robustness test

The paper conducts robustness tests by replacing the core independent variable. Fertilizer intensity is used as a substitute for fertilizer application, the core independent variable, and the model is re-estimated. The results are presented in Table 7. The robustness test demonstrates that, after replacing the core independent variable, the model results consistently show that, whether or not fertilizer use efficiency or other control variables are included, fertilizer intensity significantly and positively influences grain production at the 1% level. Additionally, fertilizer efficiency continues to significantly and positively impact grain production. With the inclusion of fertilizer efficiency and control variables, the promoting effect of fertilizer intensity on grain production diminishes. Therefore, it can be concluded that the results of the article are robust.

**Table 7:** Robustness analysis of replacing core variables.

	Estimate 1		Estimate 2		Estimate 3		Estimate 4	
	FE	2SLS	FE	2SLS	FE	2SLS	FE	2SLS
*strength*	-18.098*	112.476***	-17.784**	109.512***	-18.856*	54.767***	-18.510**	54.825***
	(-9.277)	(-12.8)	(-8.66)	(-12.251)	(-9.531)	(-12.398)	(-8.885)	(-12.492)
*eff*			-0.1143	1.2516**			-0.079	-0.0857
			(-0.157)	(-0.569)			(-0.15)	(-0.155)
*ln_GDP*					-0.383**	-0.708***	-0.377**	-0.694***
					(-0.148)	(-0.231)	(-0.154)	(-0.225)
*DR*					-0.0001	-0.0043**	-0.0001	-0.0044***
					(0.000)	(-0.002)	(0.000)	(-0.002)
*ln_irrigation*					-0.0006	0.5929***	-0.0021	0.5960***
					(-0.024)	(-0.1)	(-0.024)	(-0.102)
*ln_employee*					0.1464	1.2747***	0.1484	1.2599***
					(-0.094)	(-0.2)	(-0.095)	(-0.196)
*ln_income*					0.5703**	1.0518***	0.5387**	1.0143***
					(-0.276)	(-0.363)	(-0.251)	(-0.354)
*previous price*					0.0005	-0.0109*	0.0006	-0.0107*
					(-0.001)	(-0.006)	(-0.001)	(-0.006)
*tem*					-0.0354	-0.2162***	-0.037	-0.2179***
					(-0.031)	(-0.028)	(-0.032)	(-0.03)
*ln_precipitation*					0.024	0.8466***	0.0256	0.8628***
					(-0.066)	(-0.158)	(-0.066)	(-0.168)
*terrain*					-0.303	-0.2883***	-0.302	-0.2853***
					(0.14)	(-0.107)	(0.14)	(-0.106)
*policy*					0.0493	1.6823***	0.1284	1.7753***
					(-0.503)	(-0.639)	(-0.424)	(-0.687)
Year fixed effect	YES	YES	YES	YES	YES	YES	YES	YES
Province fixed effect	YES	YES	YES	YES	YES	YES	YES	YES
Constant	7.443***	3.749***	7.458***	3.556***	5.441**	-13.003***	5.6389**	-12.854***
	(-0.292)	(-0.447)	(-0.295)	(-0.459)	(-2.449)	(-2.725)	(-2.27)	(-2.683)
Observations	485	432	485	432	469	416	469	416
R-squared	0.351		0.358		0.428	0.739	0.43	0.739
Over-identification test		0.1993		0.2094		0.5538		0.5797
Weak instrumental variable F-value		69.0945		77.1493		22.8476		22.8093
DWH test		<1%		<1%		<1%		<1%

Note: ***, **, and * respectively indicate significance at the 1%, 5%, and 10% levels; standard errors in parentheses.

### 4.4 Heterogeneity analysis

To further analyze the heterogeneity in terms of regional location, fertilizer varieties, and crop types, the paper examines the impact of fertilizers on grain production from four perspectives: regional heterogeneity, heterogeneity in grain-producing areas, heterogeneity in fertilizers, and heterogeneity in crops.

#### 4.4.1 Heterogeneity in regions

According to the classification standards of the National Bureau of Statistics of China, it is considered that China is composed of three major regions: Eastern, Central, and Western regions. Taking into account the availability of data, this study does not currently include Hong Kong, Macau, and Taiwan. The coverage of the three major regions is outlined in Table 8, encompassing a total of 31 provinces and municipalities.

**Table 8:** Coverage of the Eastern, Central, and Western regions in China.

Region	Scope
Eastern	Beijing, Tianjin, Hebei, Liaoning, Shanghai, Jiangsu, Zhejiang, Fujian, Shandong, Guangdong, Hainan
Central	Shanxi, Jilin, Heilongjiang, Anhui, Anhui, Jiangxi, Henan, Hubei, Hunan
Western	Inner Mongolia, Guangxi, Chongqing, Sichuan, Guizhou, Yunnan, Tibet, Shaanxi, Gansu, Qinghai, Ningxia, Xinjiang

Note: Organized by the author.

Table 9 reports the impact of fertilizer usage on grain production in the eastern, central, and western regions of China. From Table 9, when introducing the interaction term between fertilizer application and fertilizer use efficiency, the p-values for the overidentification test of the instrumental variables in the eastern, central, and western regions are 0.775, 0.7662, and 0.4326, indicating that the instrumental variables are all exogenous. The corresponding F-values for the instrumental variables are 12.3966, 14.6481, and 19.6481, all exceeding 10, suggesting the absence of weak instrumental variables. The heteroscedasticity-robust DWH test results indicate that each instrumental variable is significant at the 1% level, implying that fertilizer application is an endogenous variable.

**Table 9:** Impact of regional heterogeneity (using instrumental variables).

	Eastern	Central	Western
*ln_fer×eff*	0.5735***	0.9604	0.9802***
	(-0.16)	(-0.821)	(-0.27)
*ln_fer*	0.9775***	-0.5841	-0.547
	(-0.206)	(-1.21)	(-0.37)
*eff*	-2.8912***	-5.3617	-5.1167***
	(-0.822)	(-4.775)	(-1.368)
Control variables	Control	Control	Control
Constant	-0.893	-6.8957	-16.0996***
	(-1.961)	(-4.915)	(-3.131)
Observations	128	128	160
R-squared	0.99	0.852	0.954
Year fixed effect	YES	YES	YES
Province fixed effect	YES	YES	YES
Over-identification test	0.775	0.7662	0.4326
Weak instrumental variable F-value	12.3966	14.6481	19.6481
DWH test	<1%	<1%	<1%

Note: ***, **, and * respectively indicate significance at the 1%, 5%, and 10% levels; standard errors in parentheses.

The regression results in Table 9 indicate that the interaction term between fertilizer application and fertilizer use efficiency significantly influences grain production in both the eastern and western regions, while its impact on central region grain production is not significant. Specifically, in the eastern region, the cross-term between fertilizer application and fertilizer use efficiency significantly and positively affects grain yield at the 1% level. This suggests that fertilizer use efficiency enhances the positive effect of fertilizer application on grain production, demonstrating a significant positive regulatory role. When fertilizer use efficiency increases by 1 unit, the promoting effect of fertilizer application on grain yield will increase by 0.5735 units. In the central region, the effects of fertilizer application, fertilizer use efficiency, and their interaction on grain yield are not significant. The coefficient of fertilizer application is negatively associated with grain yield, indicating that fertilizer input in the central region has gradually exceeded the optimal level, and excessive fertilizer input is detrimental to grain production. In the western region, fertilizer use efficiency mitigates the negative impact of fertilizer application on grain reduction, suggesting that improving fertilizer use efficiency in the western region can increase grain production and reduce the adverse effects of fertilizer application on grain production.

#### 4.4.2 Heterogeneity in grain-producing areas

According to the classification standards of the National Bureau of Statistics of China, the grain-producing areas in China are divided into main grain-producing areas and non-main grain-producing areas. Considering the availability of data, this study does not currently include Hong Kong, Macao, and Taiwan. The coverage of grain-producing areas is shown in Table 10, comprising a total of 31 provinces and municipalities.

**Table 10:** China’s main grain-producing regions and non-main grain-producing regions.

Region	Scope
Main Grain-Producing Regions	Heilongjiang, Henan, Shandong, Sichuan, Jiangsu, Hebei, Jilin, Anhui, Hunan, Hubei, Inner Mongolia, Jiangxi, Liaoning
Non-Main Grain-Producing Regions	Beijing, Tianjin, Shanghai, Zhejiang, Fujian, Guangdong, Hainan, Shanxi, Inner Mongolia, Guangxi, Chongqing, Guizhou, Yunnan, Tibet, Shaanxi, Gansu, Qinghai, Ningxia, Xinjiang

Note: Organized by the author.

Table 11 reports the impact of fertilizer application on different crops in the main grain-producing areas and non-main grain-producing areas. From Table 11, when introducing the interaction term between fertilizer application and grain production areas, the p-values for the over-identification test of instrumental variables for grain yield, rice yield, wheat yield, and corn yield are 0.4317, 0.3391, 0.4182, and 0.9725, respectively, indicating that the instrumental variables are all exogenous. The F-values for the weak instrument test are 45.1481, 21.1915, 52.7546, and 62.0189, all greater than 10, indicating the absence of weak instrumental variables. The robust DWH test results for heteroscedasticity show that all instrumental variables are significant at the 1% level, indicating that fertilizer application is an endogenous variable.

**Table 11:** Impact of heterogeneity in grain-producing areas (using instrumental variables).

	*Grain*	*Rice*	*Wheat*	*Corn*
*Production area×ln_fer*	-0.2220***	-0.6956	3.8545***	-0.2104
	(-0.034)	(-0.426)	(-0.33)	(-0.158)
*ln_fer*	0.5220***	-2.5833***	-4.3137***	2.2461***
	(-0.081)	(-0.98)	(-0.534)	(-0.233)
*Production area*	1.6361***	5.8710***	-19.1227***	0.4621
	(-0.192)	(-2.094)	(-1.819)	(-0.917)
*eff*	0.1411**	0.9082*	1.6374***	-0.9227***
	(-0.062)	(-0.516)	(-0.408)	(-0.181)
Control variables	Control	Control	Control	Control
Constant	-1.6048*	-15.0089**	55.0066***	3.3661
	(-0.867)	(-7.351)	(-6.518)	(-2.518)
Observations	416	400	399	409
R-squared	0.966	0.385	0.614	0.863
Year fixed effect	YES	YES	YES	YES
Province fixed effect	YES	YES	YES	YES
Over-identification test	0.4317	0.3391	0.4182	0.9725
Weak instrumental variable F-value	45.1481	21.1915	52.7546	62.0189
DWH test	<1%	<1%	<1%	<1%

Note: ***, **, and * respectively indicate significance at the 1%, 5%, and 10% levels; standard errors in parentheses.

The regression results in Table 11 indicate that the interaction term between fertilizer application and grain production areas significantly affects grain yield and wheat yield, but the impact on rice and corn is not significant. Specifically, in grain production, the interaction term between fertilizer application and grain production areas significantly and negatively affects grain yield at the 1% level, indicating that grain-producing areas inhibit the promoting effect of fertilizer application on grain production, showing a significant negative regulatory effect. When the proportion of grain-producing areas increases by 1 unit, the promoting effect of fertilizer application on grain yield will decrease by 0.222 units, suggesting that the fertilizer application in grain-producing areas has reached the optimal level, and further increasing fertilizer application will not benefit the increase in grain yield.

In wheat production, the interaction term between fertilizer application and grain production areas positively influences wheat yield at the 1% level. This suggests that grain-producing areas mitigate the negative impact of fertilizer application on wheat yield. When the proportion of grain-producing areas increases by 1 unit, fertilizer application will promote a 3.8535 unit increase in wheat yield. This indicates that in grain-producing areas, fertilizer use still has a certain promoting effect on wheat yield, and further increasing fertilizer application will enhance wheat production.

#### 4.4.3 Heterogeneity of fertilizers

Table 12 reports the effects of nitrogen fertilizer, phosphorus fertilizer, potassium fertilizer, and organic fertilizer on grain yield using the instrumental variable method. According to Table 12, after using the instrumental variable method, the p-values for the overidentification test of instrumental variables for nitrogen fertilizer, phosphorus fertilizer, potassium fertilizer, and organic fertilizer are 0.1218, 0.2227, 0.1544, and 0.2388, respectively, indicating that instrumental variables are exogenous. The corresponding F-values for the weak instrument test are 15.56863, 16.2735, 10.9059, and 14.3745, all exceeding 10, indicating the absence of weak instrumental variables. The DWH robust test results show that all instrumental variables are significant at the 1% level, indicating that fertilizer application is an endogenous variable.

**Table 12:** Impact of heterogeneity in fertilizers (using instrumental variables).

	(1)	(2)	(3)	(4)
*Nitrogen Fertilizer*	-0.0244***			
	(-0.009)			
*Phosphorus Fertilizer*		-0.0549		
		(-0.034)		
*Potash Fertilizer*			0.0494***	
			(-0.012)	
*Organic Fertilizer*				-0.0039
				(-0.007)
*eff*	-0.7989**	-0.1859	0.0224	-0.2827
	(-0.325)	(-0.262)	(-0.129)	(-0.26)
Control variables	Control	Control	Control	Control
Constant	-5.5991*	-7.7700**	-1.0878	-5.0148***
	(-3.161)	(-3.959)	(-1.77)	(-1.787)
Observations	416	416	416	416
R-squared	0.37	0.035	0.64	0.846
Year fixed effect	YES	YES	YES	YES
Province fixed effect	YES	YES	YES	YES
Over-identification test	0.1218	0.2227	0.1544	0.2388
Weak instrumental variable F-value	15.56863	16.2735	10.9059	14.3745
DWH test	<1%	<1%	<1%	<1%

Note: ***, **, and * respectively indicate significance at the 1%, 5%, and 10% levels; standard errors in parentheses.

The regression results in Table 12 indicate that nitrogen fertilizer and potassium fertilizer have significant effects on grain yield, while phosphorus fertilizer and organic fertilizer show no significant impact. This suggests that, despite being beneficial for environmentally friendly grain production, the efficacy of organic fertilizer in promoting grain yield lags behind that of nitrogen and potassium fertilizers. Specifically, the regression results in the first column of Table 12 show that nitrogen fertilizer significantly negatively affects grain yield at the 1% level. An increase of 1 unit in nitrogen fertilizer application leads to a decrease of 0.0244 units in grain yield, indicating that the optimal level of nitrogen fertilizer application has been reached, and further application may result in a reduction in grain yield. The results in column (3) of Table 12 show that potassium fertilizer significantly positively influences grain yield at the 1% level. An increase of 1 unit in potassium fertilizer application leads to an increase of 0.0494 units in grain yield.

#### 4.4.4 Heterogeneity in crops

The regression results in Table 13, columns (1) to (3), report the impact of fertilizer application on the yield of rice, wheat, and corn, respectively. According to Table 13, the p-values of the overidentification tests for the instrumental variables in columns (1) to (3) are 0.3855, 0.1571, and 0.5749, indicating that the instrumental variables are all exogenous. The corresponding F-values for the weak instrument tests are 20.3392, 47.2458, and 56.015, all exceeding 10, suggesting the absence of weak instrument issues. The robust DWH test results demonstrate that each instrumental variable is significant at the 1% level, confirming that fertilizer application is an endogenous variable.

**Table 13:** Impact of heterogeneity in crops (using instrumental variables).

	(1)	(2)	(3)
*ln_fer*	-0.4742	-6.3873***	2.0537***
	(-0.807)	(-0.838)	(-0.244)
*eff*	-0.0629	0.2894	-0.5372***
	(-0.519)	(-0.609)	(-0.172)
Control variables	Control	Control	Control
Constant	-38.9652***	46.4223***	9.3794***
	(-4.495)	(-9.443)	(-2.199)
Observations	400	399	409
R-squared	0.501	0.12	0.853
Year fixed effect	YES	YES	YES
Province fixed effect	YES	YES	YES
Over-identification test	0.3855	0.1571	0.5749
Weak instrumental variable F-value	20.3392	47.2458	56.015
DWH test	<1%	<1%	<1%

Note: ***, **, and * respectively indicate significance at the 1%, 5%, and 10% levels; standard errors in parentheses.

The regression results in Table 13, column (1), indicate that fertilizer application has no significant impact on rice yield, but the coefficient is negative, suggesting a negative effect of fertilizer application on rice production. Increasing fertilizer application is unfavorable for rice yield. In column (2), the regression results show that fertilizer application significantly negatively affects wheat yield at the 1% level. When fertilizer application increases by 1 unit, wheat yield decreases by 6.3873 units. This implies that wheat fertilizer application has reached the optimal threshold, and further application may hinder wheat yield. In column (3), the regression results demonstrate that fertilizer application significantly affects corn yield at the 1% level. An increase of 1 unit in fertilizer application leads to a gain of 2.0537 units in corn yield, indicating that fertilizer application continues to play a role in promoting corn production.

## 5. Conclusion and implications

Reducing fertilizer usage is a pivotal measure to address the contradiction between agricultural non-point source pollution and increased grain production. It represents an indispensable pathway for advancing the high-quality development of Chinese agriculture. Based on the panel data of 31 provinces from 2004 to 2019, this paper empirically analyzes the dynamic relationship between fertilizers and grain output, and draws the following conclusions: First, fertilizers are still an indispensable production factor for grain production. Fertilizer and grain output will be in a long-term "strong decoupling" and "declining decoupling" superimposed state, that is, the amount of fertilizer applied declines, grain output increases or slowly declines. Second, there is a significant substitution relationship between fertilizer usage and application rate. Reducing the application rate while enhancing fertilizer usage efficiency can not only promote stable and increased grain production but also address agricultural non-point source pollution issues. Third, the efficiency of fertilizer usage has a more pronounced impact on grain production in both the eastern and western regions. Increasing fertilizer application is not conducive to wheat yield but has a promoting effect on corn yield. However, in the main grain-producing regions, increasing fertilizer application can enhance wheat yield but is unfavorable for the overall grain production. Additionally, in comparison to potassium fertilizer, the nitrogen application rate has already exceeded the optimal level. Continuously increasing nitrogen application will be detrimental to achieving higher grain yields. To this end, the paper gives the following two insights based on the findings of the study:

Firstly, it is essential to establish a proper and environmentally friendly fertilization concept, achieving a shift from the "quantity" to the "quality" of fertilizers. It is crucial to shape farmers’ awareness of environmental protection, promote and standardize scientific fertilization practices among farmers, and encourage the adoption of efficient and environmentally friendly fertilizers. There is a need to enhance farmers’ capacity for green production, shift traditional notions and production habits that measure "grain yield" solely by "fertilizer application quantity," and fully leverage the efficacy of fertilizers.

Secondly, it is necessary to combine organic and conventional agriculture. Supporting and promoting the use of high-efficiency and environmentally friendly fertilizers is essential to improve fertilizer utilization. It is necessary to increase the research and development of high-efficiency fertilizers, with cost-saving, efficiency and arable land protection as the goal orientation. Based on the varied natural endowments of different regions and the growth characteristics of crops, maximize the utilization of existing organic fertilizer resources. Tailor the application of organic fertilizers to specific locations, aiming to achieve a dual objective of reducing chemical fertilizer usage and increasing food production.

## Supporting information

S1 Data (XLSX)
